# Glutamine metabolism in cancers: Targeting the oxidative homeostasis

**DOI:** 10.3389/fonc.2022.994672

**Published:** 2022-10-17

**Authors:** Tengfang Gong, Changbing Zheng, Xidan Ou, Jie Zheng, Jiayi Yu, Shuyu Chen, Yehui Duan, Wei Liu

**Affiliations:** ^1^ Research Center for Parasites & Vectors, College of Veterinary Medicine, Hunan Agricultural University, Changsha, China; ^2^ CAS Key Laboratory of Agro-ecological Processes in Subtropical Region, Hunan Provincial Key Laboratory of Animal Nutritional Physiology and Metabolic Process, National Engineering Laboratory for Pollution Control and Waste Utilization in Livestock and Poultry Production, Institute of Subtropical Agriculture, Chinese Academy of Sciences, Changsha, China; ^3^ College of Advanced Agricultural Sciences, University of Chinese Academy of Sciences, Beijing, China

**Keywords:** Gln metabolism, oxidative homeostasis, cancer cells, ROS, health

## Abstract

Glutamine is the most abundant amino acid in blood and tissues, and the most important nutrient except for glucose in cancer cells. Over the past years, most studies have focused on the role of Gln metabolism in supporting energy metabolism rather than maintaining oxidative homeostasis. In fact, Gln is an important factor in maintaining oxidative homeostasis of cancer cells, especially in “Glutamine addicted” cancer cells. Here, this paper will review the recent scientific literature about the link between Gln metabolism and oxidative homeostasis, with an emphasis on the potential role of Gln metabolism in different cancers. Given that oxidative homeostasis is of critical importance in cancer, understanding the impacts of a Gln metabolism on oxidative homeostasis, gaining great insights into underlying molecular mechanisms, and developing effective therapeutic strategies are of great importance.

## Introduction

**Figure 1:**
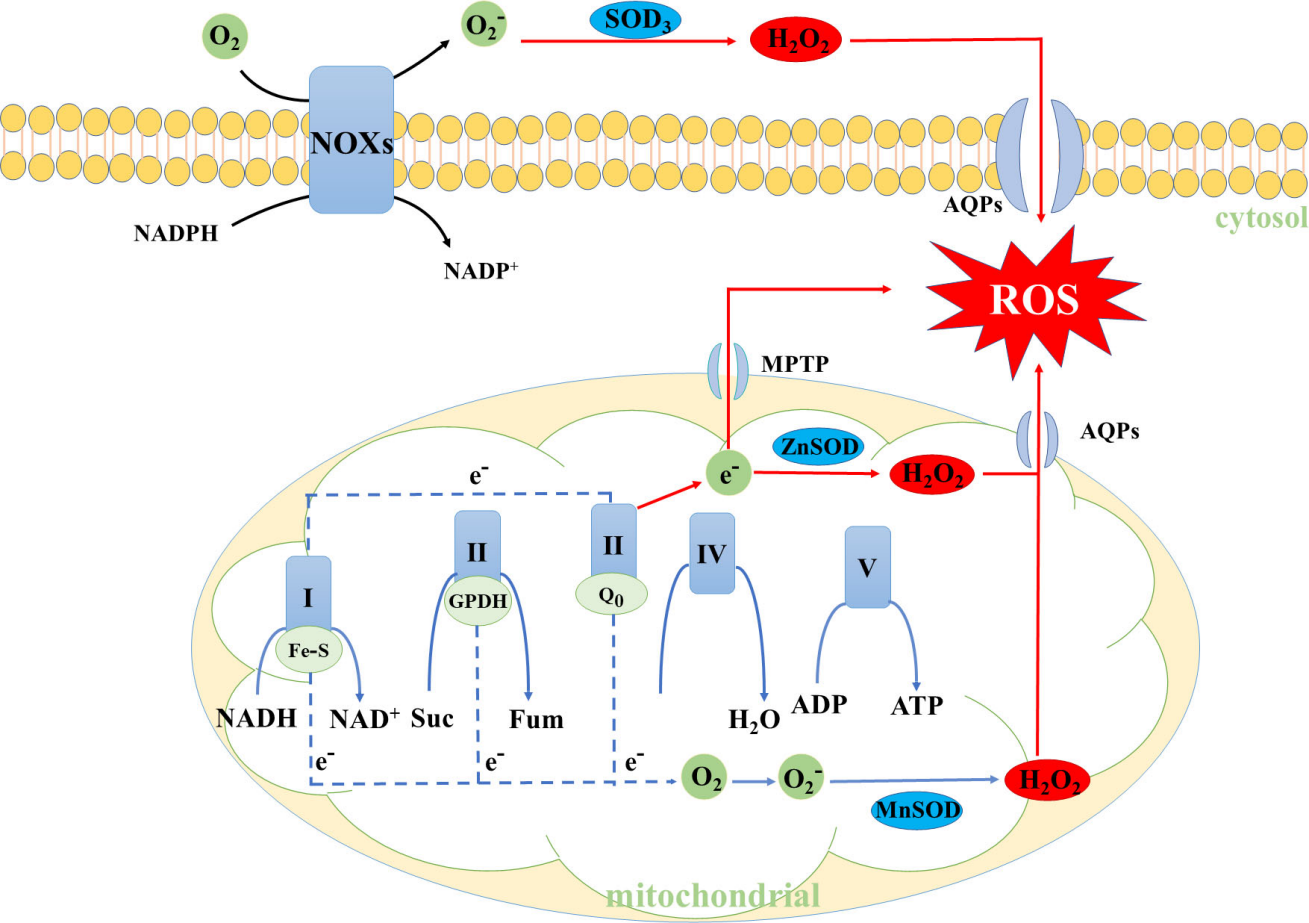
The primary generation mechanisms of intracellular ROS. SOD, Superoxide dismutase.

The reactive oxygen species (ROS), which mainly comes from the mitochondrial membrane as a byproduct of OXPHOS and nicotinamide adenine dinucleotide oxidases (NOXs), cannot avoid being produced in cellular metabolism (1–4). Cancer cells usually show higher levels of ROS, which acts as a signaling molecule in cancer, contributing to their growth and metastasis (5–8). Notably, when the levels of ROS in cancer cells are in excess, it will destroy oxidative homeostasis, subsequently damaging effects on macromolecules such as enzyme inactivation, DNA and protein damage (**Figure 1**) (9, 10). Thus, maintaining oxidative homeostasis in cancer cells is of great importance and loss of balance has profound pathophysiology consequences (11).

Glutamine (Gln), a non-essential amino acid, is essential for the survival of most cancer cells. The “Glutamine addiction” is a good description of the importance of Gln in cancer cells. When Gln is deprived of the medium, most cancer cells will be in a stagnant state or even die (12, 13). Gln metabolism, which could promote the biosynthesis of Glutathione (GSH) and nicotinamide adenine dinucleotide phosphate (NADPH), is involved in the maintenance of oxidative homeostasis in cancer cells (14). In light of the importance of Gln metabolism in oxidative homeostasis, a comprehensive understanding of the mechanics is vital for developing of tumor therapies. This review will elaborate on the functions of Gln and its products in the oxidative homeostasis of cancer cells, including roles in the biosynthesis of GSH and NADPH, and will explore the roles of Gln metabolism in different cancers *via* regulating oxidative homeostasis.

## Gln metabolism in oxidative homeostasis

**Figure 2:**
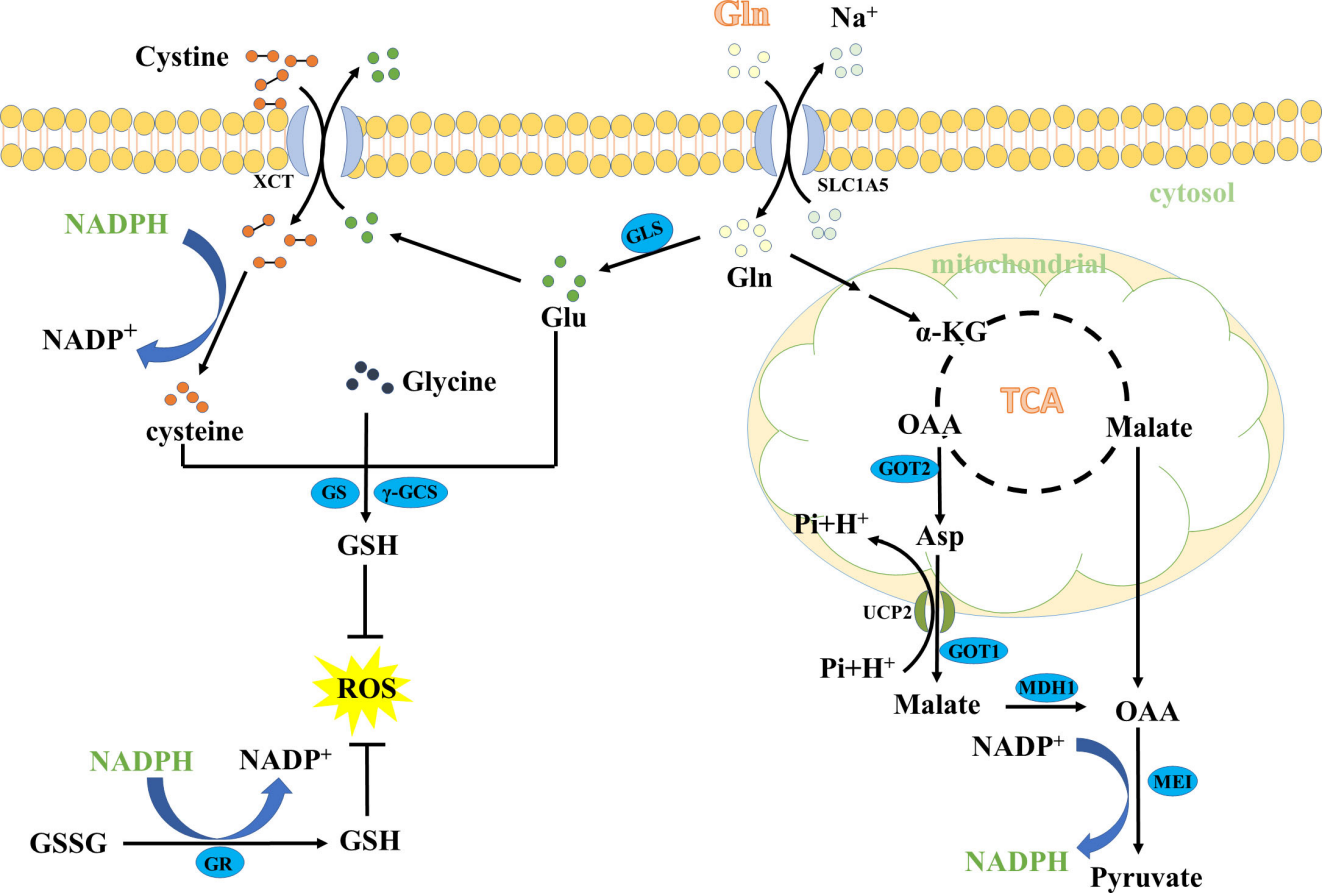
The key role of glutamine in GSH and NADPH biosynthesis. MDH1, malate dehydrogenase 1; SLC1A5, the solute carrier family 1, member 5.

The Gln metabolism could maintain oxidative homeostasis through many pathways. One of the most important pathways is through promoting the biosynthesis of GSH. Glutamate (Glu), cysteine, and glycine are required amino acids for *de novo* biosynthesis of GSH (15–17). Notably, the conversion of Gln to Glu is required to maintain the large intracellular pools of Glu (13). Typically, Gln is first taken in by cancer cells through the transporters (such as ASCT2, ATB^0,+^, System L, System A), and then converted to Glu (18–20). The Gln-converted Glu subsequently generates GSH in two ways (**Figure 2**). On the one hand, Glu can be polymerized with cysteine in an ATP-dependent manner to form γ-glutamylcysteine, and further condense with glycine to produce GSH (21–24). On the other hand, Glu is transported *via* cystine/glutamate antiporter xCT (also commonly known as SLC7A11) to the extracellular for exchanging cystine and a subsequent conversion of cystine to cysteine through a NADPH-consuming reduction reaction. The generated cysteine is subsequently used to form GSH (25, 26). GSH is a powerful reducing agent that acts as a free radical scavenger. Maintaining high levels of GSH in cancer cells can eliminate excessive ROS and detoxify xenobiotics to avoid oxidative damage.

Besides the role in the *de novo* biosynthesis of GSH, Gln also contributes to NADPH production. First, Gln enters the TCA cycle, and directly generates malate, or indirectly forms malate from the conversion of Asp *via* the Asp transporter mitochondrial uncoupling protein 2 (UCP2) and the enzymes aspartate transaminase (GOT1) and malate dehydrogenase 1 (MDH1). Then, malate crosses the mitochondrial membrane to the cytoplasm and is further catalyzed to pyruvate *via* the malic enzyme 1 (ME1), accompanied by reducing NADP to NADPH (27–29). Importantly, NADPH can reduce glutathione disulfide (GSSG) to GSH, an essential cofactor maintaining the reduced form of GSH (30, 31). On the other hand, NADPH can reduce cystine to cysteine for *de novo* biosynthesis of GSH (32, 33). Therefore, NADPH plays a role in the production of GSH, thus contributing to the maintenance of redox balance.

Overall, the Gln metabolism in this review refers to the metabolic pathway of the formation of GSH and NADPH from Gln, which could help maintain oxidative homeostasis of cancer cells and hence promote their progression.

## The potential role of Gln metabolism in different cancers

**Figure 3:**
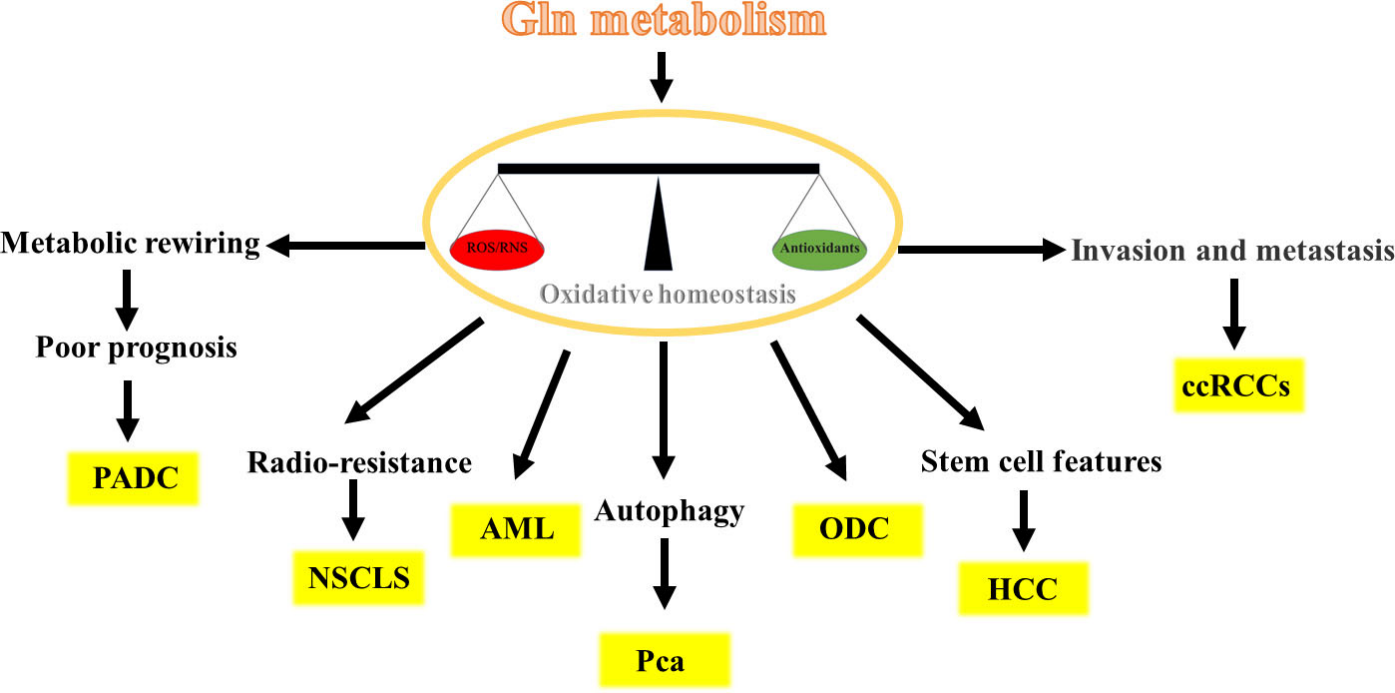
Different potential roles of Gln metabolism in different cancer cells.

Gln metabolism has different potential roles in different cancer cells by maintaining oxidative homeostasis and is crucial for cancer development. In the following sections, we describe in detail the role of Gln metabolism in different cancer cells (**Figure 3**).

### Pancreatic ductal adenocarcinoma

Pancreatic ductal adenocarcinoma (PADC) is common malignant and poor prognosis tumors with a 5-year survival rate of approximately 10% in the USA (34–36). Multiple pieces of evidence have demonstrated that Gln metabolism implicates the progression of PADC induced by internal or external factors. For instance, Gln-metabolism is required for the hypoxia-inducible factor-2a-promoted PDAC progression (37). Moreover, the oncogenic *KRAS*-triggered PDAC growth is accompanied by the metabolic rewiring of Gln metabolism, which fulfills the NADPH need and balances cellular oxidative homeostasis (29). Similar increased production of Gln-derived NADPH is observed upon oxidative stress, accompanied by the survival and growth of PADC (38). These findings present us with intriguing evidence that the Gln-derived NADPH may positively associate with the poor prognosis of PDAC (39, 40). In addition, it has been demonstrated that PADC development-required NADPH strongly relies on Gln metabolism rather than on the pentose phosphate (PP) pathway. Evidence to support this hypothesis is that the Gln-derived NADPH markedly decreased after the knockdown of GOT1 or ME1 in PADC cells, which caused a significant increase in the ratio of GSSG/GSH, whereas glucose deprivation or knockdown of the limiting enzyme G6PD in the PP pathway had only a modest impact on NADPH (29, 41). Further evidence comes from the finding that the knockdown of UCP2 (the Asp transporter) decreased Gln-derived NADPH levels and increased ROS levels in PDAC cells, thus suppressing PDAC cell growth (42). Taken together, Gln-derived NADPH is required for the progression of PADC, and targeting this distinct pathway represents a novel prognostic biomarker and therapeutic target for patients with PDAC.

### Acute myeloid leukemia

Several recent studies have demonstrated that Gln metabolism is implicated in the progression of acute myeloid leukemia (AML), as evidenced by exerting antileukemic effects (43–47). However, most of these studies focus on the role of Gln in supporting energy metabolism rather than maintaining oxidative homeostasis. Therefore, to better understand the role and regulatory mechanism of Gln metabolism in oxidative homeostasis of AML, one study using a FLT3-mutated AML cell model found that impaired Gln metabolism by FLT3 inhibitors could lead to depletion of GSH and accumulation of mitochondrial reactive oxygen species (mitoROS), subsequently leading to apoptosis of AML cell (48). A similar reduction of GSH levels and elevation of mitoROS and apoptosis were observed when AML cell lines were treated with the glutaminase inhibitor CB-839 for 24 h, which led to an inhibition of Gln metabolism (49).. These findings suggest that depletion of GSH is a universal consequence of inhibition of Gln metabolism in AML. In addition, inhibition of Gln metabolism makes AML cells susceptible to adjunctive drugs that further impair oxidative homeostasis. For example, combination of arsenic trioxide (ATO) and homoharringtonine (HHT) (the potent inducers of mitoROS) with CB-839 the exacerbates accumulation of mitoROS and apoptosis, which leads to complete cell death in AML cell lines, primary AML patient samples and *in vivo* mouse models of AML (49). Overall, Gln metabolism is implicated in promoting the development of AML, and the use of a Gln metabolism inhibitor in combination with drugs that further induces mitoROS and apoptosis may represent an effective and widely applicable therapeutic strategy for treating multiple types of AML.

### Non-small cell lung cancer

In general, radiotherapy alone or in combination with chemotherapy and adjuvant durvalumab are mainly therapeutic methods for patients with locally advanced non-small cell lung cancer (NSCLC) (50, 51). However, after radiotherapy, the patient is prone to loco-regional recurrence, which remains a major clinical challenge for the cure for NSCLC (52–55). Existing evidences has linked Gln metabolism to the radio-resistance in NSCLC. For instance, a recently published article showed that the liver kinase B1-deficient NSCLC cells strongly depend on Gln-derived GSH to reduce ionizing radiation-derived ROS generation and to alleviate radiation-derived cytotoxic effects under radiotherapy. On the contrary, inhibition of Gln metabolism using knockdown of GLS could impair oxidative homeostasis, resulting in radio-sensitization of NSCLC (56). Another study also showed that the knockdown of GLS could increase response to radiotherapy of NSCLC by 30% *in vitro* and *in vivo* (57). Consistently, other studies also show that inhibition of Gln metabolism could suppress the GSH levels and enhanced radiosensitivity of NSCLC (58–60). These results indicate that NSCLC relies on Gln-derived GSH to maintain oxidative homeostasis to resist radiotherapy. All in all, inhibition of Glu metabolism may serve as a potential therapeutic strategy to cure this highly refractory subgroup of NSCLC patients.

### Hepatocellular carcinoma

Liver cancer stem cells (CSCs), a subset of liver cells with stem cell features, are considered to be responsible for hepatocellular carcinoma (HCC) recurrence, metastasis, and chemoresistance (61, 62). These cells are heavily implicated in the Wnt/β-catenin pathway which is identified as one of the most frequent events occurring in CSCs (63, 64). It has been recognized that Gln metabolism is strongly correlated with Wnt/β-catenin pathway activation, contributing to liver carcinogenesis, hampering patient prognosis, and treatment stratification (65–67). Up to further investigations, the researchers found that the stemness properties in HCC were regulated by Gln metabolism through a ROS/Wnt/β-catenin signaling positive-feedback loop. More specifically, Gln metabolism could maintain low amounts of ROS and Wnt/β-catenin activation, which causes accumulation of β-catenin in the cytoplasm and then promotes the translocation of β-catenin to the nucleus. β-catenin in the nucleus activates the expression of CSC markers, such as NANOG, OCT4, KLF4, SOX2, and c-MYC and other Wnt target genes in HCC cell lines, thus promoting the progression of HCC (68). Interestingly, this study has also shown that the activated Wnt/β-catenin pathway *via* its agonist SKL2001 could upregulate the mRNA and protein levels of GLS1, and then promote Gln metabolism, which means that activated Wnt/β-catenin pathway could promote GLS expression with positive feedback (68). A similar study has shown that the high expression of GLS1 in HCC had a markedly shorter overall survival time than its low expression (69). Taken together, Gln metabolism can increase the stemness properties in HCC through activating ROS/Wnt/β-catenin pathway, and targeting Gln metabolism, especially GLS1, may be a therapeutic target for the elimination of CSCs.

### Prostate cancer

Prostate cancer (Pca) treatments, such as radiation, chemotherapy, and hormone therapy, can induce autophagy that improves therapeutic resistance (70–72). Existing evidence has linked the Gln metabolism to autophagy through oxidative homeostasis in Pca. For instance, a recently published article showed that the radio-resistant Pca cells strongly rely on Gln metabolism to maintain oxidative homeostasis. However, Pca cells could trigger autophagy upon Gln withdrawal and do not exhibit significant radio-sensitization (73). Upon further investigations, the researchers found that the ionizing radiation-derived ROS can induce autophagy as a stress response of Pca cells, but it is neutralized by GSH and NADPH produced by Gln metabolism. When blocking Gln metabolism, Pca cells could activate the ATG -mediated autophagy as a survival strategy to withstand radiation-induced damage due to GSH depletion and ROS accumulation (73, 74). Consistently, other studies also confirmed that autophagy inhibition increases ROS production in Pca cells (75–77). Overall, Gln metabolism affects the autophagy of Pca cells by affecting the level of ROS.

### Kidney cancer

Kidney cancer, the ideal model of metabolic reprogramming among all cancers, has been duly named as a “Metabolic Disease” (78–81). There is growing evidence that clear cell renal cell carcinoma cells (ccRCCs) are Gln-addicted that is reprogrammed to feed an intrinsic antioxidant system (82–84). For instance, combined proteomics and metabolomics studies have shown that the ccRCC largely uses Gln to feed the GSH/GSSG antioxidant system to attenuate oxidative stress, rather than to generate energy and cellular components through the TCA cycle (85). To further confirm the role of Gln as a source for the GSH pathway, absolute quantitative GSH and GSSG levels in cells grown with and without Gln were compared. The result showed that GSH and GSSG levels were markedly reduced in the Gln-depleted group, which confirms the necessity of Gln for maintaining oxidative homeostasis of ccRCCs (85). Similar findings were obtained in another study, showing that inhibition of Gln metabolism *via* CB-839 led to decreased GSH/GSSG ratio, and furtherly increased oxidative stress and ccRCCs apoptosis (86). In addition, an interesting study shows that the suppression of fatty acid metabolism by inhibition of β-oxidation lead to the RCC cells dependent on the Gln-GSH pathway to prevent lipid peroxidation and ferroptosis (87). Notably, high GSH levels have proven to be a key feature of high-grade, high-stage and metastatic ccRCCs (81, 88). All in all, these data suggest that Gln-dependent antioxidant effects may provide ccRCCs with a critical mechanism for their survival.

### Oligodendroglioma

In general, Gln is an antioxidant defense only in Gln addicted cancers, but not in all cases. Oligodendroglioma cells lack Gln synthetase (a marker of Gln-addicted cancers), but are independent of extracellular Gln (thus are not Gln addicted) (89, 90). However, a previous study showed that small amounts of extracellular Gln are sufficient for oligodendroglioma cells growth. Gln starvation does not significantly affect the cell content of anaplerotic substrates, but causes a significant decrease in the intracellular content of GSH in oligodendroglioma cells (91). This result means that Gln addiction and Gln roles as antioxidants are not correlated. In addition, Gln starvation causes hindrance of the Wnt/β-catenin pathway and protein synthesis attenuation in oligodendroglioma cells, which means that Gln may stimulate Wnt/beta-catenin pathways by ROS levels to affect the activity of cells, as in HCC (68, 91).

## ROS production and ferroptosis

**Figure 4:**
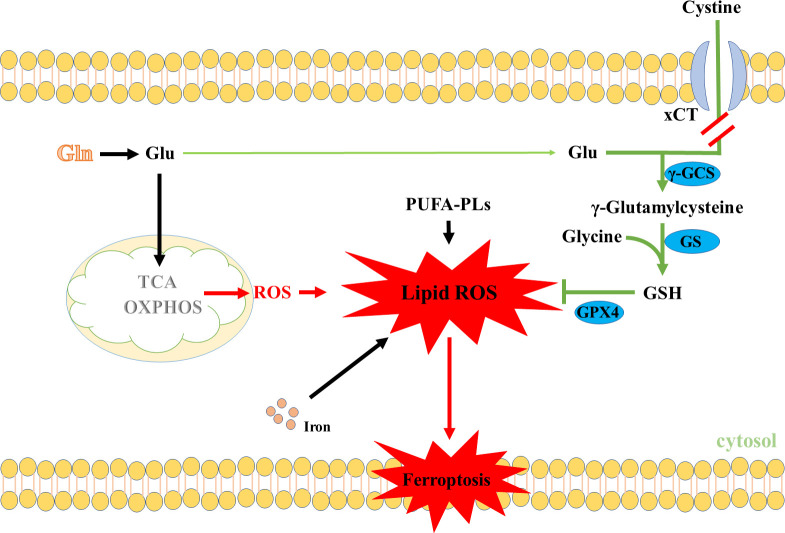
Gln metabolism promotes ROS production through the TCA cycle. PUFA-PLs, Polyunsaturated fatty acid chain(s).

In light of the findings mentioned above, it would seem reasonable to expect that Gln metabolism plays an important role in maintaining ROS levels in cancer cells. However, we noted that most of the above-mentioned studies have mainly focused on the effects of Gln metabolism on maintaining oxidative homeostasis of cancer cells, whereas these effects were not suitable for every situation. Some studies have shown that the anaplerotic role of Gln metabolism in replenishing the TCA cycle intermediates could enhance ROS production under the blocking of GSH synthesis (92–94). For instance, a recently published article showed that Gln metabolism was crucial to maintaining cystine starvation-induced mitochondrial membrane potential (MMP) hyperpolarization, accompanied by an increase in electron transfer chain (ETC) activity and lipid ROS generation to promote ferroptosis (95). In support of this notion, data from various studies showed that inhibiting the glutaminolysis can suppress TCA cycle and MMP hyperpolarization, and reduce lipid ROS production, thus enhancing ferroptosis resistance (95–98). Similarly, various studies showed that inhibiting xCT activities could suppress Gln-derived Glu export and enhance Glu to replenish the TCA cycle intermediates (99–101). Therefore, it has been theorized that inhibition of xCT activities could promote Glu to replenish the TCA cycle intermediates, which could promote ROS production (102) (**Figure 4**). All in all, increasing ROS levels by Gln metabolism under blocking of GSH synthesis promoted ferroptosis, which may provide a novel treatment guideline for ferroptosis-based tumor therapy.

## Therapeutic strategies targeting Gln metabolism in cancer

The demonstration of the link between Gln metabolism and oxidative homeostasis of cancer has prompted research into strategies to target Gln metabolism to damage oxidative homeostasis of cancer. In this regard, GLS inhibitors aimed at decreasing Gln metabolism and impairing oxidative homeostasis are attracting increasing clinical interest. Many small molecules have been assayed to block GLS isoenzymes after the first attempt and failure to use 6-diazo-5-oxo-L-norleucine (DON) as an anti-cancer drug (103, 104). The bis-2-(5-phenylacetamido-1,2,4-thiadiazol-2-yl) ethyl sulfide (BPTES) and CB-839 are the specific inhibitors most frequently (86). Notably, CB-839 is currently being administered to humans in phase 1 clinical trials for some types of cancers (49, 103–106).

However, because of the plasticity of adaptive metabolic reprogramming in cancer cells, successful single treatments against cancers are scarce (4, 107–109). Therefore, some specific inhibitor of Gln metabolism has reached better results in sensitizing cancer cells to other treatments (110). Targeting Gln metabolism combined with drugs that are strong inducers of mitochondrial ROS, is widely used for treating multiple cancers (**Table 1**). For instance, dihydroartemisinin cooperatively induces excessive intracellular ROS resulting in profound apoptosis when combined with CB-839 in HCC (111). In a similar study, Gregory et al. demonstrated that a combination of GLS inhibition with ATO or HHT showed great activity against AML (49). Preclinical studies have also reported a benefit when combined with Gln metabolism inhibitors and radiotherapy. For example, the inhibitor CB-839 increased GSH depletion, and enhanced the radiation sensitivity of lung tumor cells xenografts in mice (57). Interestingly, one recent study showed that the combination of Gln metabolism inhibitors with radiotherapy could activate the ATG5-mediated autophagy of Prostate cancer, and proposes a strategy that a combination with autophagy inhibition and the blockade of Gln metabolism makes Pca radio-sensitization (73, 74, 122). Notably, the chemotherapy and/or radiation can also cause cellular damage in normal organs and tissues by generating free radicals (123). Antioxidants such as vitamins, minerals, and polyphenols can quench ROS activity alleviate the adverse effects of chemotherapy and/or radiotherapy (124, 125). Combining inhibition of Gln metabolism with antioxidant supplementation may enhance the chemotherapy and/or radiation sensitivity while preventing cellular damage of normal organs and tissues, which may be an effective strategy for the treatment of cancer. However, it remains controversial whether antioxidants affect treatment outcomes or whether antioxidants ameliorate adverse effects induced by chemotherapy and radiotherapy, which needs further investigations in the future (126). In conclusion, combination therapy, including inhibitors of Gln metabolism, may be a promising strategy for cancer cells.

**Table 1:** Combined treatments: targeting glutaminolysis in combination with drugs that unbalance mitochondrial redox state.

Type of cancer	Target Gln metabolism		Combined treatment	Drug mechanism	References
	Site	Type of inhibition			
**AML**	GLS	CB-839	ATO; HHT	Inducing excessive ROS	(49)
**HCC**			Dihydroartemisinin	Inducing excessive ROS	(111)
**NSCLC**			Radiotherapy	Radiosensitization	(56)
**Pca**		GLS siRNA silencing	ATG5 siRNA silencing; Radiotherapy	Inhibition of autophagy; Radiosensitization	(74)
**PDAC**			ß-lapachone	Inducing excessive ROS	(112)
**GBM**			ATO, H2O2	Inducing excessive ROS	(113)
**TNBC**		Compound 968	CQ	Inhibition of autophagy; inducing excessive ROS	(114)
**NSCLC**					(115)
**LCLC**			Apigenin	Inducing excessive ROS	(116)
**GBM**	GLS2	GLS2 overexpression	ATO; H2O2	Inducing excessive ROS	(113)
**BC**	SLC1A5	V9302	anti-PD-1 monoclonal antibody (mAb)	Enhancing antitumor immunity	(117)
**HNSCC**		Cetuximab	Dichloroacetate	Inducing excessive ROS	(118, 119)
**CC**	SLC1A5/GDH1	CB-839/R162	CAI	Inducing excessive ROS	(120)
**BC**	/	Glutamine deprivation	Vorinostat	Inducing excessive ROS	(121)
**CC**			

BC, Breast cancer; CAI, Carboxyamidotriazole; CQ, chloroquine; CC, Colon cancer; GBM, glioblastoma; GDH1, glutamate dehydrogenase 1; HNSCC, head and neck squamous cell carcinoma; LCLC, large cell lung carcinoma; TNBC, triple-negative breast cancer; V9302, glutamine metabolism inhibitor.

## Conclusion

The antioxidant capacity of tumor cells is required for rapidly proliferating and aggressive cancer cells to adapt to hypoxia and excessive ROS levels. The literature reviewed here suggests that Gln has been established as an important factor in maintaining the oxidative homeostasis of cancer cells. Targeting Gln metabolism impaired oxidative homeostasis of cancer cells and may provide effective approaches for therapies against cancer. In addition, more research is urgently needed to implement multiple synergistic targeting (including Gln metabolism inhibitors) to block tumor proliferation and increase cancer cells’ sensitivity of cancer cells to other therapies. Future studies on Gln metabolism in maintaining oxidative homeostasis may provide novel and effective therapeutic strategies to treat a subset of cancer patients.

## Author contributions

Conceptualization, YD, WL, and TG; writing—original draft preparation, TG, CZ, XO, JZ, JY, and SC; writing—review and editing, YD and WL; visualization, YD. All authors have read and agreed to the published version of the manuscript.

## Funding

This study was jointly supported by the Natural Science Foundation of the Hunan Province, China (2021JJ30335, 2021JJ20044), the National Natural Science Foundation of China (U19A2037), the Changsha Natural Science Funds for Distinguished Young Scholar (kq2009020), Young Elite Scientists Sponsorship Program by CAST (2020-2022QNRC003), the Natural Science Foundation of Guangxi Province (2020JJA130102), China Agriculture Research System of MOF and MARA (CARS-35), and the ‘Strategic Priority Research Program’ of the Chinese Academy of Sciences (XDA24030204), the Scientific Research Fund of Hunan Provincial Education Department, China (21A0141).

## Conflict of interest

The authors declare that the research was conducted in the absence of any commercial or financial relationships that could be construed as a potential conflict of interest.

## Publisher’s note

All claims expressed in this article are solely those of the authors and do not necessarily represent those of their affiliated organizations, or those of the publisher, the editors and the reviewers. Any product that may be evaluated in this article, or claim that may be made by its manufacturer, is not guaranteed or endorsed by the publisher.
